# Dysfunctional BLK in common variable immunodeficiency perturbs B-cell proliferation and ability to elicit antigen-specific CD4^+^ T-cell help

**DOI:** 10.18632/oncotarget.3577

**Published:** 2015-03-14

**Authors:** Ewoud B. Compeer, Willemijn Janssen, Annet van Royen-Kerkhof, Marielle van Gijn, Joris M. van Montfrans, Marianne Boes

**Affiliations:** ^1^ Department of Pediatric Immunology, Laboratory of Translational Immunology, Wilhelmina Children's Hospital, University Medical Center Utrecht, The Netherlands; ^2^ Department of Medical Genetics, Laboratory of Translational Immunology, Wilhelmina Children's Hospital, University Medical Center Utrecht, The Netherlands

**Keywords:** common variable immunodeficiency, B-lymphoid tyrosine kinase, BLK, spleen tyrosine kinase, antigen presentation

## Abstract

Common Variable Immunodeficiency (CVID) is the most prevalent primary antibody deficiency, and characterized by defective generation of high-affinity antibodies. Patients have therefore increased risk to recurrent infections of the respiratory and intestinal tract. Development of high-affinity antigen-specific antibodies involves two key actions of B-cell receptors (BCR): transmembrane signaling through BCR-complexes to induce B-cell differentiation and proliferation, and BCR-mediated antigen internalization for class-II MHC-mediated presentation to acquire antigen-specific CD4^+^ T-cell help.

We identified a variant (L3P) in the B-lymphoid tyrosine kinase (BLK) gene of 2 related CVID-patients, which was absent in healthy relatives. BLK belongs to the Src-kinases family and involved in BCR-signaling. Here, we sought to clarify BLK function in healthy human B-cells and its association to CVID.

BLK expression was comparable in patient and healthy B-cells. Functional analysis of L3P-BLK showed reduced BCR crosslinking-induced Syk phosphorylation and proliferation, in both primary B-cells and B-LCLs. B-cells expressing L3P-BLK showed accelerated destruction of BCR-internalized antigen and reduced ability to elicit CD40L-expression on antigen-specific CD4^+^ T-cells.

In conclusion, we found a novel BLK gene variant in CVID-patients that causes suppressed B-cell proliferation and reduced ability of B-cells to elicit antigen-specific CD4^+^ T-cell responses. Both these mechanisms may contribute to hypogammaglobulinemia in CVID-patients.

## INTRODUCTION

Common variable immunodeficiency (CVID) is the most common primary immunodeficiency (PID), with an estimated prevalence of 1 in 25.000-50.000 adult Caucasians [1]. CVID patients are characterized by defective generation of high affinity antibodies by B-cells and therefore suffer from recurrent infections of the respiratory- and intestinal tract [1-3].

The vast majority of B-cells recirculate through the blood and the follicular regions of the lymphoid tissues. These quiescent B-cells require sustained expression of functional B-cell receptors (BCRs) to transduce antigen-independent tonic signals for their survival. B-cells may capture encountered antigen with their specific BCR that is composed of the Igα/Igβ dimer. Productive BCR-antigen engagement initiates a signaling cascade that starts with the activation of tyrosine kinases from the Src family. Src kinases phosphorylate the ITAM motif of Igα and Igβ. For example, the Src kinase family member B-lymphoid tyrosine kinase (BLK) phosphorylates the ITAM motif of Igα [4]. This leads to the recruitment and subsequent activation of Spleen tyrosine kinase (Syk) [5]. Ultimately this leads to functional B-cell activation and clonal expansion. For the development of high affinity antigen-specific antibodies, in addition BCR expression and signaling is necessary to internalize antigen into endosomes for processing by endosomal proteases. Antigen-derived peptides thereby generated are subsequently presented as antigen-specific peptide/class II MHC complexes to acquire antigen-specific CD4^+^ T-cell help in form of cytokines and co-stimulatory molecules, such as IL-4 and CD40L respectively. Therefore it is not surprising that in patients showing characteristics of CVID, monogenetic defects are found in genes relevant to BCR signaling or the elicitation of CD4^+^ T-cell help, including CD19 [6], CD20 [7], CD81 [8], or CD27 [9], CD40L [10] and ICOS-L [11].

Using our recently developed Primary Immunodeficiency (PID) targeted Next-Generation Sequencing-based approach [12], we screened patients with CVID of unknown origin for (putative) PID-associated gene variants. Here we report a Leucine to Proline replacement at position 3 in B-lymphoid tyrosine kinase (BLK) in 2 patients with CVID. BLK was initially thought to be expressed solely in B-lineage cells [13], but is now known to be also expressed in both human and mouse pancreatic β-cells [14], in murine plasmacytoid dendritic cells [15], and is required for the development of T-cells and IL-17-producing γδ T-cells in mice [16]. BLK belongs to the Src family of tyrosine kinases that phosphorylate Igα subunit of BCR signalling complex [4, 17]. We sought to clarify BLK function in human B-cells and its association to CVID. To this end, we researched the effect of L3P-BLK gene variant expression on BLK function and B-cell function in primary CVID-patient B-cells and immortalized B-LCLs.

Unlike in mice, where to a certain extent functional redundancy exists between Src kinases [18-20], in human B-cells the L3P-BLK mutation had functional consequences to BCR signaling. We propose that BLK is pivotal to normal B-cell function as antigen presenting cells. Moreover, BLK disease variants may contribute to CVID disease pathology by perturbing B-cells to proliferate and adequately elicit CD4^+^ T-cell help, known to support B-cells class-switching of Ig heavy chain regions and eventually the sufficient production of high affinity IgG and IgA antibodies.

## RESULTS

### Case report of index patient and family

The index patient presented in our hospital at age 7, with a history of severe recurrent pulmonary infections from the age of 8 months onward, requiring frequent hospitalizations. Laboratory investigations showed hypogammaglobulinemia with persistent low levels of IgG (5g/L), low IgA levels (0.42g/L), and relatively normal IgM levels (0.39g/L) (Table 1). The patient was vaccinated according to the national vaccination program in the Netherlands, however showed insufficient vaccination responses for Haemophilus Influenza B (Hib), Meningococci type C, and pneumococcal polysaccharide antigens 4-6 weeks after re-vaccination. Based on these findings the patient was diagnosed with CVID [1] and treated with monthly infusion of intravenous immunoglobulin (Ig), and antibiotic prophylaxis.

To study potential causes of the antibody deficiency, flow cytometric analysis was performed on peripheral blood of the patient. This showed normal numbers of monocytes (0,4×10^9^/L), lymphocytes (2,47×10^9^/L) and NK cells (11%), but slightly reduced B-cell numbers (Table 2). Within the B-cell compartment, IgG memory B-cells were in the low normal range (2.6% of B-cells), but IgM- (5.6%) and IgA-positive memory B cells (0.2%) were significantly reduced [21]. There were no signs for autoimmunity or lymphoproliferative disease.

The patient's father had a disease history comprising of recurrent respiratory tract infections and episodes of bacteriaemia upon small skin lesions. Moreover, he had relative low IgM (0,28g/L) and IgG (6,65g/L) levels (shown in Table 1). In contrast to both the index patient and his father, the mother, sister and another brother showed no clinical symptoms related to antibody deficiency. The mother was excluded from functional experiments, because she was treated with immunosuppressive therapy for recently diagnosed ulcerative colitis.

**Table 1:** Serum antibody titers of L3P-BLK carrying individuals in comparison to age-matched controls

Immunoglobulins		patient (L3P-BLK)	reference values	father (L3P-BLK)	reference values
IgM-total	g/L	0.39	0.28-1.9	0.28	0.40-2.3
IgA-total	g/L	0.42	0.54-2.5	1.2	0.7-4.0
IgG-total	g/L	5	5.20-14.3	6.65	7.00-16.0
IgG1	g/L	3.4	3.5-9.1	4.1	4.9-11.4
IgG2	g/L	0.62	0.85-3.30	1.91	1.50-6.40
IgG3	g/L	0.34	0.20-1.04	0.20	0.20-1.10
IgG4	g/L	0.02	0.03-1.58	0.05	0.08-1.40
**Post-vaccination: serum antibody titers**		**patient (L3P-BLK)**	**father (L3P-BLK)**	**reference values**	
Meningococci C	mg/ml	0.33	<0.24	>1mg/ml	
HiB-BL	μg/mL	0.32	0.50	>1μg/ml	
PnPS1-BL	μg/mL	0.16	0.38	>1μg/ml*	
PnPS3-BL	μg/mL	0.22	3.2	>1μg/ml*	
PnPS4-BL	μg/mL	0.10	0.049	>1μg/ml*	
PnPS5-BL	μg/mL	0.26	0.072	>1μg/ml*	
PnPS6B-BL	μg/mL	0.27	0.061	>1μg/ml*	
PnPS7F-BL	μg/mL	0.72	>40	>1μg/ml*	
PnPS9V-BL	μg/mL	0.43	0.18	>1μg/ml*	
PnPS14-BL	μg/mL	1.4	4.5	>1μg/ml*	
PnPS18C-BL	μg/mL	4.9	0.051	>1μg/ml*	
PnPS19F-BL	μg/mL	1.2	0.38	>1μg/ml*	
PnPS23F-BL	μg/mL	0.13	0.082	>1μg/ml*	

* >6 years of age, a normal response is defined as IgG reponses of >1μg/ml in at least 8/11 pneumococcal polyssacharide serotypes measured.

### Screening of Common Variable Immunodeficiency-associated candidate genes uncovers a L3P point mutation in the BLK gene

**Fig.1:**
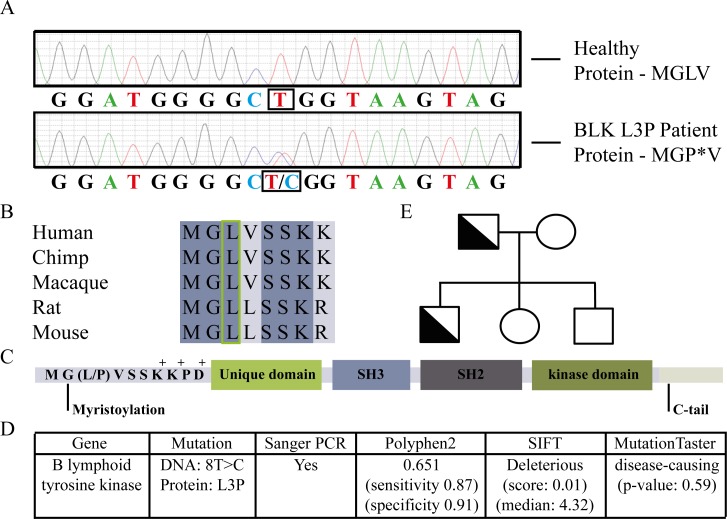
Classification of the novel L3P mutation in B lymphoid tyrosine kinase of Common Variable Immunodeficiency (CVID) patients A. Confirmation of heterozygous mutation L3P in B lymphoid tyrosine kinase of CVID patient by Sanger PCR. B. The mutated Leucine indicated is an amino acid conserved amongst species. C. The mutation occurs at third position, between a myristoylated Glycine residue and a charged amino acid cluster. D. Three bioinformatics models predict a deleterious effect of the mutation on BLK function. E. Family tree showing CVID diagnosed L3P-BLK carrying patients (father and son) and non-carrying healthy family members.

Regular genetic analysis of the index patient revealed no mutations in known CVID-associated genes. Hence, we exploited our recently developed Primary Immunodeficiency (PID)-targeted Next-Generation Sequencing-strategy based on 170 PID-related (IUIS) and 350 candidate PID-genes. This strategy allows detection of point mutations with a sensitivity and specificity >99% in covered regions [12], and revealed that both CVID patients but not their healthy relatives had a heterozygous point mutation (NM_001715.2 c.8T>C) in the BLK gene. No mutations were found in the other known or candidate PID-genes present on the chip [12]. The point mutation in BLK was subsequently confirmed by Sanger sequencing, as shown in Figure 1A. The L3P-BLK gene variant is unique to these CVID patients, with healthy family members not carrying the mutation (Figure 1E) and it is not present in the dbSNP, or Dutch population-specific GoNL databases. Moreover the residue changed by the mutation is a highly conserved Leucine at the third position to a Proline (Figure 1B and 1C), predicted to be potentially damaging by PolyPhen-2, SIFT and Mutation Taster (Figure 1D).

**Fig.2:**
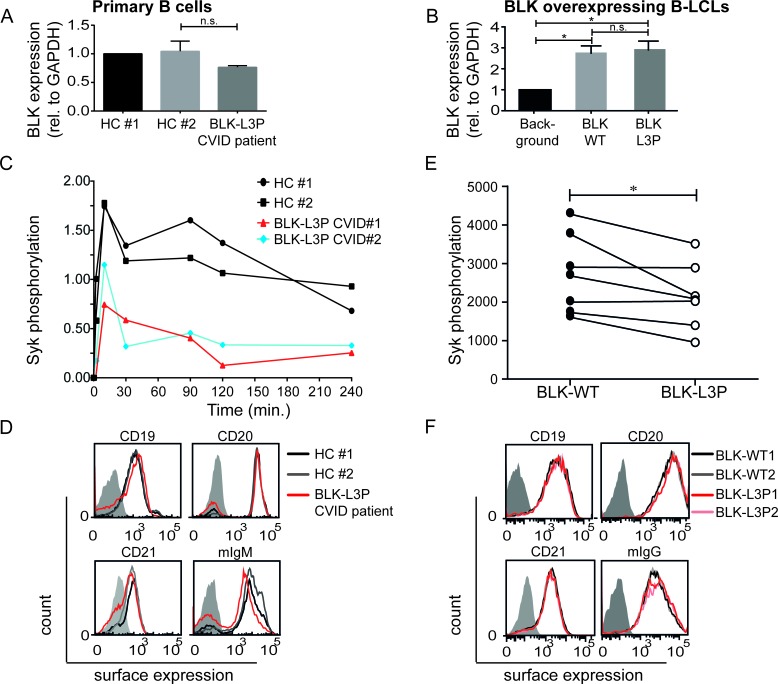
Reduced Spleen tyrosine kinase (Syk) phosphorylation upon B cell receptor crosslinking of CVID (L3P-BLK) patient CD20 B cells Quantitative PCR demonstrates that heterozygous L3P-BLK CVID patient comparable levels of BLK mRNA (p=0.25) in A. CD19^+^ B cells relative to healthy controls or B. BLK overexpressing B-LCLs. Data of 3 independent experiments represented as mean +/− SEM. C. B cell receptors are crosslinked by incubation of PBMCs from CVID (L3P-BLK) patient or healthy controls with excess amounts of anti-IgM and IgG F(ab')2 fragments for the indicated duration (0, 2, 10, 30, 90, 120, and 240 minutes). At the end of incubation the cells are fixed and surface-stained with CD20 antibody. Subsequently, the cells are permeabilized with methanol and stained for intracellular presence of phosphorylated Syk (pSyk), followed by flow cytometric analysis on live (FSC/SSC) CD20^+^ B cells. Representative of 3 independent experiments, presented as mean fluorescent intensity levels of phosphorylated Syk over time in CD20^+^ B cells of healthy controls (Black), CVID (L3P-BLK) patient (Red), and CVID patient #2 (Blue) also heterozygous for L3P-BLK mutation. D. Expression of B cell (co)receptor molecules CD19, CD20, CD21, and membrane-bound IgM as B cell receptor (mIgM) in CD20^+^ B cells of healthy controls (Black and Grey) and CVID (L3P-BLK) patient (Red). Representative of 3 independent experiments. Filled grey graphs are non-stained negative controls. E. LMP2a driven phosphorylation of Syk in B-LCLs overexpressing either L3P- or common BLK variant in simultaneously executed experiments. F. Expression of B cell (co)receptor molecules CD19, CD20, CD21, and membrane-bound IgM as B cell receptor (mIgM) in B-LCLs overexpressing L3P-BLK (Red) or common BLK variant (black/grey). Representative of 3 independent experiments. Grey filled graphs are non-stained B-LCLs. *P-value <0.05, **P-value <0.01, Two-tailed Wilcoxon-signed rank test.

We determined by quantitative analysis that BLK RNA expression is not significantly different in human peripheral blood CD19^+^ B-cells of healthy individuals and our CVID patients carrying L3P-BLK gene variant (Figure 2A), but is absent in monocyte-derived dendritic cells (DCs) and human CD4^+^ T-cells in circulation (our data not shown). Additionally, we seem to observe similar levels of BLK protein in human peripheral blood CD19^+^ B-cells of healthy individuals and our index CVID patients (supplemental Figure 1A). Thus, L3P mutation appears to affect BLK function. Together this data prompted us to perform functional studies.

### Reduced Syk phosphorylation upon BCR crosslinking in L3P-BLK patient B-cells

In B-cells, BLK protein associates with Syk and Igα upon activation [22, 23]. The latter is phosphorylate by BLK upon antigen-induced BCR-crosslinking [4]. However, experiments executed in COS cells using an overexpressed mutated L3C-BLK earlier demonstrated that BLK-mediated Igα phosphorylation was blocked [17], suggesting that in analogy, L3P-BLK in human cells may have a functional defect at phosphorylation. We tested this possibility by measuring Syk phosphorylation, the immediate downstream consequence of Igα phosphorylation. We induced BCR-crosslinking by addition of anti-IgM and IgG-F(ab')2 fragments, fixed PBMCs at indicated time points, and measured levels of phosphorylated (p)Syk in CD20^+^ B-cells by flow cytometry as previously performed by others [5]. Instead of anti-IgM/G antibodies, anti-IgM/anti-IgG F(ab')2 fragments were used. This approach ensures specific BCR targeting and eliminated non-specific binding with Fc receptors. In healthy controls, BCR crosslinking induced increased amounts of pSyk within 2 minutes, which corroborates with previous data [5]. In contrast, in L3P-BLK carrying CVID patient B-cells pSyk levels were induced later and remained at 50% lower levels in comparison to primary B-cells of healthy individuals (Figure 2C). Surface expression of B-cell (co)receptor complex molecules CD19, CD20, CD21, and membrane-bound IgM, was similar on B-cells from individuals carrying L3P- and common BLK variant (Figure 2D). Thus, BCR-mediated signaling rather than B-cell (co)receptor complex components expression levels appear affected in L3P-BLK carrying B-cells of our CVID patients.

### Reduced Syk phosphorylation upon BCR-crosslinking in L3P-BLK-overexpressing B-cell lines

In B-LCLs, that are immortalized by Epstein-Bar virus (EBV), EBV-derived Latent Membrane Protein 2a (LMP2A) drives activation of α- and β-chains of the BCR [24]. Similarly as the BCR, LMP2a associates with Src family of tyrosine kinases [25]. This capacity of B-LCLs allowed us to study the effect of L3P-BLK variant expression on B-cell signaling and function. To this end, we drove overexpression of either L3P- or common BLK variant in 3 separately derived B-lymphoblastoid cell lines (B-LCLs) by retroviral transduction. To enable comparison, we selected clones within each B-LCL cell line with similar overexpression levels of either L3P- or common BLK protein as determined by quantitative PCR (Figure 2B) and Western blot (Supplemental Figure 1B).

To address whether L3P mutation in BLK is solely responsible for the reduced levels of Syk phosphorylation observed in the primary CD19^+^ B-cells of our CVID patients (Figure 2C), we analyzed B-LCLs overexpressing BLK and L3P-BLK for relative levels of pSyk similarly as described above. In analogy of the L3P-BLK primary B-cell data, also B-LCLs that overexpressed L3P-BLK exhibited reduced Syk phosphorylation without affecting expression levels of the B-cell (co)receptor molecules CD19, CD20, CD21, and membrane-bound IgG (Figure 2E and 2F). Thus, the L3P-BLK variant suppresses BCR crosslinking-induced Syk phosphorylation, when compared to the common BLK protein.

### L3P-BLK variant negatively affects tonic signaling-dependent B-cell proliferation

**Fig.3:**
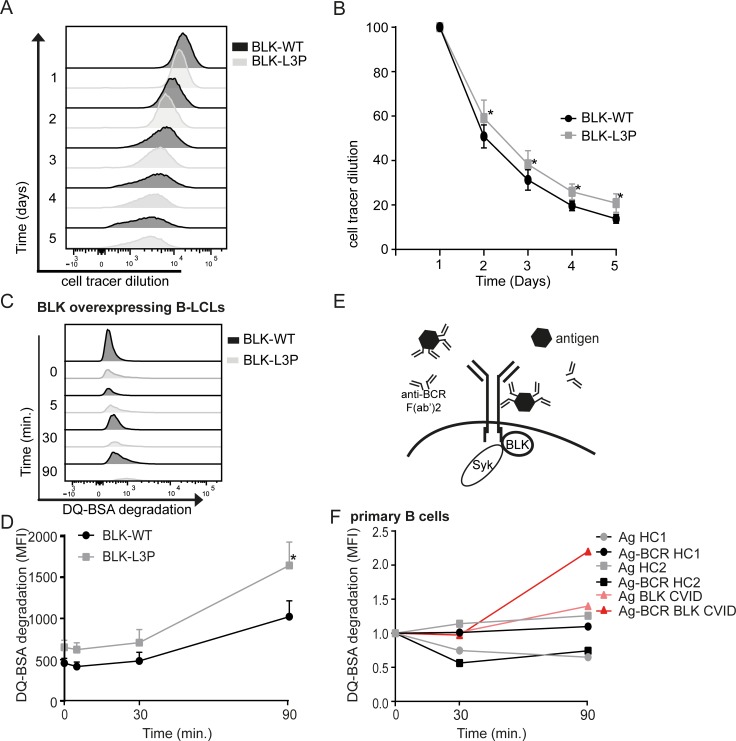
L3P-BLK has diminished ability to transmit tonic and ligand-induced B cell receptor signals A and B. B-LCLs overexpressing either L3P- or common BLK variant are stained with cell tracer violet and B cell proliferation was determined by dilution of cell tracer violet MFI measured by flow cytometry each day. Data of 3 independent experiments, represented as mean +/− SEM. C. DQ-BSA becomes fluorescent when cleaved. DQ-BSA degradation was measured by flow cytometry as MFI increase. D and E. Streptavid-modified DQ-BSA is complexed in 4:1 ratio with biotinylated anti-IgM and anti-IgG to target to BCR. These anti-BCR/DQ-BSA complexes are administered to B-LCLs overexpressing L3P- or common BLK variant, or F. in primary CD19^+^ B cells derived from healthy volunteers (squares and circles) or BLK-L3P carrying CVID patients (triangles). Representative (F) of Data (D) of 3 independent experiments, represented as mean +/− SEM. *P-value <0.05, **P-value <0.01, Two-tailed Wilcoxon-signed rank test.

B-cell proliferation requires tonic BCR signaling via Src family kinases to which BLK belongs [4]. We considered this finding in light that our L3P-BLK CVID index patient shows reduced numbers of circulating IgD^−^CD27^+^IgM and IgD^−^CD27^+^IgA memory B-cells (Table 2). Therefore, we investigated whether and to which extend L3P-BLK may affect B-cell proliferation. We stained B-LCLs overexpressing either the L3P-BLK or common BLK variant with Cell Tracer Violet at day 1, to assess the B-cell proliferation rate. During 4 days, cell tracer violet fluorescence was monitored using flow cytometry (Figure 3A). Already after the first day and continuing until the last day, B-LCLs overexpressing L3P-BLK had a small but significant delay in B-cell proliferation when compared to common BLK carrying B-LCLs (Figure 3B). All together, these data support that L3P-BLK has less proficiency than the common BLK variant to transduce tonic BCR signaling towards B-cell proliferation, which may contribute to the reduced B-cell number phenotype in the L3P-BLK CVID patient.

**Table 2:** Lymphocytes in peripheral blood of L3P-BLK carrying individual in comparison to age-matched controls

Lymphocyte subset		patient (L3P-BLK)	reference values
CD3^+^	(% within Ly)	76	57-76
CD3^+^CD4^+^ T-cells	(% within Ly)	46.1	29-46
CD3^+^CD8^+^ T-cells	(% within Ly)	24.5	19-34
CD19^+^ B-cells	(% within Ly)	11	12-26
NK cells	(% within Ly)	11	6-21
IgM^+^IgD^+^CD27^−^ naïve B-cells	(% within B-cells)	74	52-73
CD27-CD21^int^CD38^++^CD10^+^IgM^++^ trans. B-cells	(% within B-cells)	9	2.9-23.8
IgD^−^CD27^+^IgM memory B-cells	(% within B-cells)	5.6	6.5-22.2
IgD^−^CD27^+^IgG memory B-cells	(% within B-cells)	2.6	1.5-8.8
IgD^−^CD27^+^IgA memory B-cells	(% within B-cells)	0.2	1.3-6.1

### B-cell receptor endosomal routing upon antigen binding is altered in B-LCLs that overexpress L3P-BLK compared to the common BLK variant

While superfluous in tonic BCR signaling, Syk kinase function is considered essential for the propagation of antigen-induced B-cell signaling (reviewed in [23]). To investigate whether the L3P-BLK defect to elicit Syk phosphorylation propagates to downstream defects, we determined the fate of BCR-targeted antigen upon endocytosis, using DQ-BSA complexed to anti-IgG F(ab')2 fragments (Figure 3E). DQ-BSA is a model antigen that becomes fluorescent upon proteolytic degradation in acidic late endosomal compartments. After BCR-mediated uptake of anti-IgG/DQ-BSA complexes in B-LCLs, we observed accelerated degradation of DQ-BSA in L3P- than common BLK expressing B-LCLs (Figure 3C and 3D). Similarly, our CVID patient B-cells showed faster degradation of specifically BCR-targeted DQ-BSA in comparison to non-targeted DQ-BSA or CD19^+^ B-cells derived from healthy individuals (Figure 3F).

Internalization of BCR complexes upon crosslinking with anti-aIgG antibodies seems unaffected in B-LCLs expressing either BLK variant (supplemental figure 2), which corroborates with unaltered expression of B-cell (co)receptor complex components on B-cell surface (Figure 2F). All together, the L3P-BLK variant when seems to direct the sorting of BCR-internalized antigen/IgG complexes towards proteolytic active, degradative endosomal compartments.

### L3P-BLK variant obstructs B-cell receptor-mediated antigen presentation to antigen-specific CD4^+^ T cells

**Fig.4:**
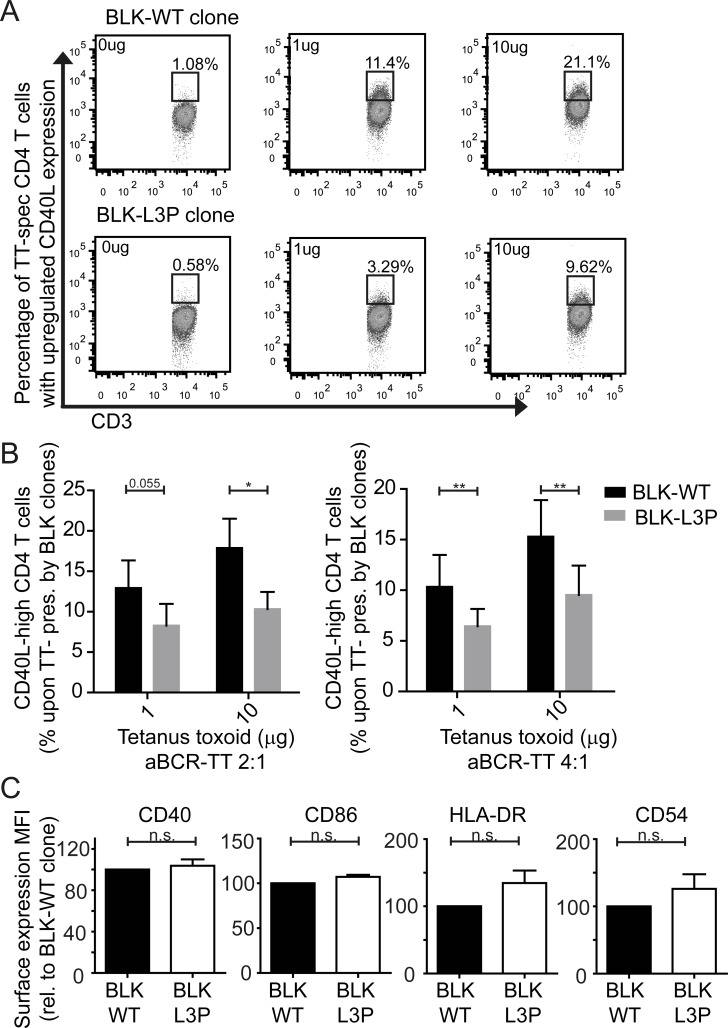
Decreased capacity of L3P-BLK to facilitate B cell receptor-mediated HLA-DR/peptide presentation to CD4 T-cells Tetanus toxoid (TT) protein is complexed with anti-IgG F(ab')2 fragments in ratio 1:2 or 1:4 for 6 hours at 4C (using streptavidin/biotin conjugation). Paired human B-LCLs expressing common or L3P-BLK variant are loaded (4hrs, 37°C) with 0, 1, or 10 μg anti-IgG-TT complexes. Next, human TT peptide/MHCII-specific CD4^+^ T-cells are added for co-culture with the B-LCLs (1:1 ratio, O/N, 37°C). B-LCL mediated activation of antigen-specific CD4^+^ T-cells was measured by analysis of induced CD154 (CD40L) expression. A. CD40L staining in absence of exogenous antigen is set as background levels for both B-LCLs expressing common BLK (upper panel) or L3P-BLK (lower panel). Representative of 3 independent experiments. B. L3P-BLK expressing B-LCL cells (grey) are less able to induce activation of antigen-specific CD4^+^ T-cells (CD40L high phenotype) compared to common BLK-expressing B-LCL cells (black). Experiments performed on B-LCLs of three independent donors, in at least three independent experiments. Data represented as mean +/− SEM. C. Expression of L3P-BLK does not affect expression of class II MHC molecules (HLA-DR) and co-stimulatory molecules (CD40, CD86, and CD54) in B-LCLs. Data of 3 independent experiments, represented as mean +/− SEM. *P-value <0.05, **P-value <0.01, Two-tailed Wilcoxon-signed rank test.

Could the rapid degradation of antigen-BCR complexes modulate the ability of L3P-BLK B-cells to function as antigen presenting cells? The generation of high-affinity IgG and IgA antibodies requires class II MHC-mediated antigen presentation by B-cells to invoke T-cell help and CD40 binding by CD40L upregulation on antigen-triggered T-cells [26]. Indeed, Syk signaling participates in BCR-mediated antigen processing and presentation of human B-cells [27, 28]. Considering both L3P-BLK CVID patients are hypogammaglobulinemic and have low post-vaccination titers elicited by T-cell- dependent antigens, we hypothesized that L3P-BLK protein may be less capable to facilitate class II MHC antigen presentation by B-cells, when compared to the common BLK protein. To address this question, we generated Tetanus Toxoid (TT)-IgM/G immune complexes by incubating biotinylated anti-IgM/anti-IgG F(ab')2 fragments to streptavidin-conjugated TT in 2:1 or 4:1 (*w:w*) ratio overnight (Figure 3B) [29]. We administered BCR-targeted TT-Ig complexes to paired B-LCL lines expressing either L3P-BLK or the common BLK for 4 hours (37°C), followed by overnight co-culture with TT-specific CD4^+^ T-cells. We determined stimulation levels of TT-specific CD4^+^ T-cells by measure of upregulated CD40L, using flow cytometry (Figure 4A). The fraction of CD4^+^ T-cells expressing high levels of CD40L increases in antigen dose-dependent manner by both L3P- or common BLK variant expressing B-LCLs. However, antigen-driven activation of the TT-specific CD4^+^ T-cells is significantly suppressed in co-cultures using B-LCLs expressing L3P-BLK compared to common BLK variant (Figure 4A, 4B). These data support that L3P-BLK obstructs antigen-specific B-cell mediated antigen presentation to CD4^+^ T-cells. Considering that the recruitment of CD4^+^ T-cell help in form of CD40-CD40L engagement is required to generate high affinity antibodies, these data clarify an unexpected role for B-cell expressed L3P-BLK in the elicitation of CD4^+^ T-cell help, with relevance to CVID disease pathology.

After 4 hours of incubation of TT-anti IgG F(ab')2 complexes with B-LCLs overexpressing L3P- or common BLK variant, we measured the surface expression levels of CD40, CD86, class II MHC (HLA-DR), and CD54 (ICAM-1). Surface expression of these markers was comparable, as determined by flow cytometry (Figure 4C). This unaltered display of HLA-DR and co-stimulatory molecules suggest that the L3P mutation in BLK does not affect overall antigen-induced BCR-crosslinking dependent B-cell activation. Thus, the L3P-BLK variant obstructs BCR-signaling and sorting of BCR-internalized antigen towards endosomal compartments conductive to Class II MHC-mediated antigen presentation. These data clarify a previously unrecognized role of human BLK through Syk signaling in the support of antigen processing and peptide/Class II MHC presentation by B-cells.

## DISCUSSION

The selection and expansion of antigen-specific B-cells to become functional Ig-secreting plasma cells and generate memory B-cells requires BCR-signaling and antigen-specific CD4^+^ T-cell help, most notably through CD40L-CD40 engagement [30-32]. Molecules involved in the Ig isotype class-switching and eventual production of large amounts of high affinity antibodies include RAG [33], AID [34], and UNG [35], and additionally the following B- and T-cell receptor signaling molecules are involved: CD20 [7, 36], CD21 [37], CD81 [8], ICOS [38], and CD40L [39]. Mutations affecting genes encoding these proteins are found in human patients that suffer from primary immunodeficiencies, including CVID [2]. We here report the functional analysis of a CVID-associated variant in the protein BLK, a Src-kinase family member that serves early downstream of the BCR. We describe a defect in BCR-triggering-induced Syk phosphorylation by the CVID-associated L3P-BLK variant. Analysis of this CVID-related BLK mutant moreover allowed us to clarify a role for BLK in B-cell proliferation and BCR-mediated antigen presentation to elicit activation of antigen-specific CD4^+^ T-cells in humans.

As member of the Src kinase family, BLK shares a conserved tyrosine kinase domain with other family members. While mouse-based experiments suggested that the catalytic activity of Src kinases is redundant [18-20], we found that in human B-cells this is not the case. Specificity is supported by tissue-specific expression and subcellular localization. Myristoylation, at the second amino acid of BLK, is necessary for its membrane localization and kinase activity. Exchange of the third amino acid of BLK for a cysteine has earlier been shown to abrogate its ability to phosphorylate Igα [17]. Hence, our identified mutation of the third amino acid of BLK into Proline may cause defective myristoylation and thereby affect localization of BLK to lipid bilayers. However, the retroviral construct we used to overexpress BLK and L3P-BLK in B-LCLs has incorporated an additional myristoylation-tag distal from the L3P mutation, thereby relieving the requirement of the myristoylation motif around the L3P mutation. Thus, while we did find a functional defect in B-LCLs overexpressing L3P-BLK, the defect in patient B-cells may be even more severe due to obstruction of myristoylation-mediated subcellular membrane localization. Of note, besides myristoylation, additional motifs support the anchorage of Src kinases to membranes, including palmitoylation [17, 40]. In Src, and probably BLK, several basic amino acids appear to interact with inner leaflet membrane phospholipids that are acidic [40]. Considering that electrostatic interactions are sensitive to disruption, supporting the possibility that a L3P mutation could have effects on localization and thereby function. Attempts to address this possibility directly, by visualization of the subcellular localization of BLK and L3P-BLK in primary B-cells from patients and healthy controls, were unfortunately unsuccessful due to the relative small cytosol volume present in primary B-cells.

In mice, functional redundancy between Src kinase family members is apparent as a single Src kinase member-knockout mice has no or subtle deficiencies [18-20]. Only when several Src family members are deleted, in double or triple knock outs, major defects are observed; SRC/YES and SRC/FYN are lethal whereas HCK/FGR double knock-out mice are immune-compromized [18]. In the human, Src-kinase family members are less superfluous [41]. Especially BLK seems to be non-redundant in function, as BLK is the only Src kinase family member that is able to phosphorylate and subsequently associate with co-transfected Igα and Igβ chimeras *in vivo* [4]. We believe this to be the reason that the CVID-associated BLK mutation has functional consequences.

Diminished B-cell proliferation and T-cell help is associated with reduced numbers of class-switched memory B-cells and defective production of high affinity antibodies, as showed for CD20 [2, 36], CD21 [37], CD81 [8], ICOS [11], and CD40L [42] deficient CVID patients. In addition, selective CVID patient T-cells have a reduced T-cell responses to tetanus toxoid, even though primary allo-stimulation of the same T-cells was normal in CVID patients [43]. Moreover, reduced CD4^+^ T-cell numbers are reported in several CVID patients. All these data support that defective elicitation of CD4^+^ T helper cell help may contribute or even cause pathology in a subset of CVID patients. In line with this, our CVID patients that also show reduced numbers of class-switched memory B-cells and defective production of high affinity antibodies carry a L3P-BLK variant that distort BCR signaling required for B-cell proliferation and recruitment of T-cell help. We propose that dysfunctional BLK variant underlies CVID disease pathology by perturbing B-cell proliferation and elicitation of antigen-specific CD4^+^ T-cell help. Further research should be aimed to determine the proportion of CVID patients that harbor defects in BLK or other early B-cell activation-related signaling molecules, and how gene defects overall relate to distinct B-cell functions as antigen presenting cells and Ig-secreting plasma cells.

## MATERIALS AND METHODS

### Patients and healthy donors

The index patient, his parents, and his brother and sister were included in this study. Adult volunteers were healthy employees of the University Medical Center Utrecht. This study was approved by the institutional review board, and informed consent was obtained.

### Targeted Next-Generation Sequencing

The Next-Generation Sequencing is targeting 170 PID-related (IUIS^2^) and >350 putatively PID-related genes^9^. We used both targeted array-based and in-solution enrichment combined with a SOLiD sequencing platform and bioinformatics analysis, as described previously [12]. Subsequently, the selected variant was validated with Sanger sequencing. Amplicons were bidirectly sequenced with the Big Dye Terminator version 3.1 cycle sequencing kit and an ABI 3730 DNA Analyzer (Life Technologies). Sequences were compared with reference sequences by using Mutation Surveyor (SoftGenetics). The prevalence of the BLK gene variant was determined in the dbSNP and GoNL exome databases.

### B-cells overexpressing B-Lymphoid tyrosine Kinase variants

The CVID-associated mutation of BLK was inserted in pWZL-Neo-Myr Flag-BLK (Plasmid 20430, Addgene) by site-directed mutagenesis according to manufacturers protocol (Qiagen) using primers (Sigma-Aldrich): BLK Fwd1: CACCTGGATGAAGACAAGCA and BLK Rev1: CCTTCCGACCCTGTGATCTA. Packaging cells (Phoenix-Ampho) were transfected with gag-pol (pHIT60), env (pCOLT-GALV), and pWZL-Neo-Myr Flag-BLK wildtype or disease-associated variant, using Fugene6 (Promega). The produced virus particles were applied to freshly thawed B-Lymphoblastoid Cell Lines from 4 different healthy donors. After 1 week of selection, B-LCLs were used in experiments.

### Quantitative PCR

Freshly isolated PBMCs or cultured B-LCLs overexpressing BLK disease-associated or wildtype variant were lysed and total mRNA was isolated using Tripure isolation reagent (Roche Diagnostics) according to the manufacturer's instructions. RNA concentrations were measured by spectrophotometer and equalized for all samples prior to reverse transcription using an iScript cDNA synthesis kit (Biorad). Primers were mixed with IQ SYBR green supermix (BioRad). The detection run started at 95°C for 10 min, followed by 45 cycles of 95°C for 15s and 60°C for 1 min. Assays were performed in duplicate or triplicate as 15μl reactions in 96well plates using C1000 Thermal Cycler (BioRad). Results were normalized to the endogenous GAPDH and Actin mRNA. The following primers were used: GAPDH Forward 5′-GTCGGAGTCAACGGATT-3′; GAPDH Reverse 5′-AAGCTTCCCGTTCTCAG-3′; Actin Forward 5′-CATGTACGTTGCTATCCAGGC-3′; Actin Reverse 5′-CTCCTTAATGTCACGCACGAT -3; BLK Forward 5′-CACCTGGATGGAAGACAAGCA-3′; BLK Reverse 5′-CCTTCCGACCCTGTGATCTA-3′ (All Sigma-Aldrich).

### Flow cytometry and functional assays

Isolate PBMCs by Ficol-plaque and let them rest for at least 2hours at 37C. Stimulate rested PBMCs or equal amount of B-LCLs (BLK-wt and L3P variant) for 0, 4, 10, 30, 90, 120, and 240 min. with goat anti-human IgM and IgG F(ab')2 fragments (5μg/ml). This is followed by fixation with 1.3% EM grade paraformaldehyde (Electron Microscopy Technologies) for 5 min. at RT. These cells were washed and taken up in FACS buffer (PBS complemented with 1% Bovine Serum Albumin (BSA, Roche) and 0.1% Sodium Azide). Extracellular CD20 (Pacific Blue, Biolegend) and IgM or IgG (PE, Fab fragments, Invitrogen) were stained (RT, 30 min.) prior to permeabilization with ice-cold methanol (4C, 5 min.). Cells are rehydrated and washed with PBS+ 1% BSA, and phosphorylated Syk was stained (PECy7, cl17A1P-ZAP70, BD Bioscience, RT, 20min.)

Goat anti-human IgG and IgM F(ab')2-fragments (Invitrogen) were conjugated to EZ-link Sulfo-NHS Biotin according to manufactures protocol (Thermo Scientific). Purified Tetanus Toxoid (RIVM, the Netherlands) or DQ-BSA (Life Technologies) was conjugated to Lightening-link streptavidin according to manufactures protocol (Novus Biologicals). Biotinylated anti-IgM or anti-IgG F(ab')2 fragments are complexed overnight to streptavadin-conjugated DQ-BSA or Tetanus Toxoid in 2:1 or 4:1 (*w:w*) ratio.

For CD4^+^ T-cell activation assay, these complexes were incubated for 4 hours to PBMCs or B-LCLs. Followed by overnight incubation with tetanus toxoid-specific CD4^+^ T-cell clones. Antigen-specific CD154 expression on CD3^+^ CD4^+^ T-cells was determined by staining with CD154 (Pacific Blue, Biolegend), CD3 (APC, BD Bioscience), and CD4 (FITC, eBioscience).

For antigen degradation assay, anti-IgM/G DQ-BSA complexes are administered to B-LCLs expressing either L3P- or common BLK variant and put on ice at indicated timepoints. BCR-targeted antigen destruction was determined by Flow cytometric analysis of the MFI emitted by processed DQ-BSA per B-LCL.

BCR complex molecules and co-stimulatory molecules were determined by flow cytometric analysis upon staining PBMCs or B-LCLs with following antibodies: CD19, CD21, CD81, CD40, and CD54 (all from BD Bioscience); CD20, CD86, HLA-DR, CD154, streptavadin-APC (all Biolegend).

### B-cell proliferation assay

B-LCLs proliferation was determined by the dilution rate of Cell Tracer Violet (Invitrogen). To this end, 1×10^6 B-LCLs expressing either BLK variants are stained with 2μM Cell Tracer Violet for 10 minutes at 37C. Staining and excess Cell tracer Violet is removed by spinning B-cells down in 5x volume Fetal Calf Serum (FCS). Cells were maintained in a humidified incubator at 37 °C with 5% CO_2_ for 4 days in RPMI 1640 medium with 1% (v/v) PenStrep (Invitrogen), 1% (v/v) GlutaMAX (Invitrogen), and 10% (v/v) FCS. Mean Fluorescent Intensity was determined on the Flow cytometer at day 1, 2, 3, 4.

### Statistical analysis

Flow cytometry data were collected on FacsCanto II (Becton Dickinson) and analyzed with BD FACSDiva v6.1.3 and Flowjo 7.6 software (Treestar). All data were statistically analyzed and plotted with GraphPad Prism® 5 software (GraphPad Software, Inc, La Jolla, California).

## SUPPLEMENTARY MATERIAL FIGURES



## Abbrevaitons

BCR: B cell receptor
B-LCL: B lymphoblastoid cell line
BLK: B lymphoid tyrosine kinase
CVID: common variable immunodeficiency
EBV: Epstein-Barr Virus
ICOS: Inducible T cell costimulator
Ig: Immunoglobulin
ITAM: immunoreceptor tyrosine-based activation motif
LMP2a: Latent Membrane Protein 2a
Syk: Spleen tyrosine kinase
TT: Tetanus Toxoid
