# Supplementary material for: Dysfunctional BLK in common variable immunodeficiency perturbs B-cell proliferation and ability to elicit antigen-specific CD4+ T-cell help
Source: Oncotarget. 2015 Mar 14;6(13):10759–71. doi: 10.18632/oncotarget.3577 (PMC4484417; doi:10.18632/oncotarget.3577)
Supplement: Supplementary file 1 [file oncotarget-06-10759-s001.pdf]

Dysfunctional BLK in common variable immunodeficiency perturbs B-cell proliferation and ability to elicit antigen-specific CD4^{+} T-cell help

Supplementary Material

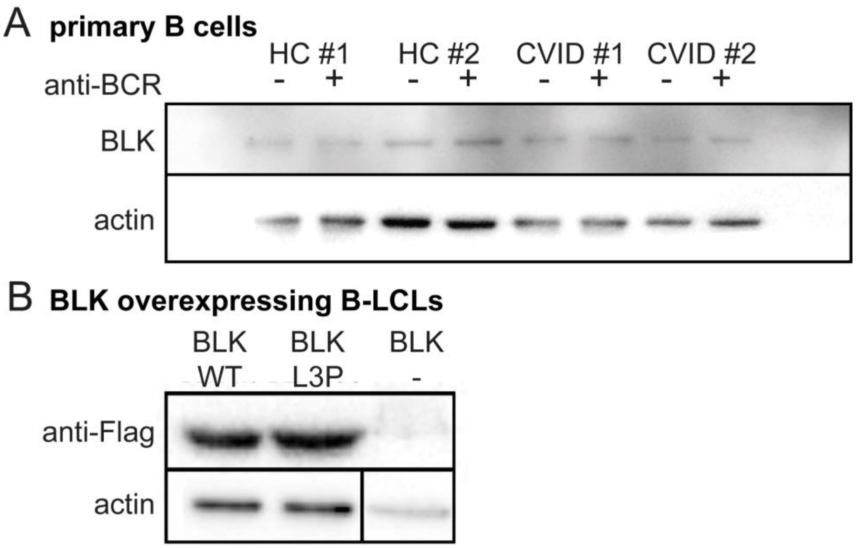

Figure S1: BLK protein expression. (**A**) Expression of endogenous BLK protein in primary CD19^{+} B cells of healthy or L3P-BLK CVID patients, or (**B**) common and L3P variant construct-derived BLK protein, as determined by western blots.

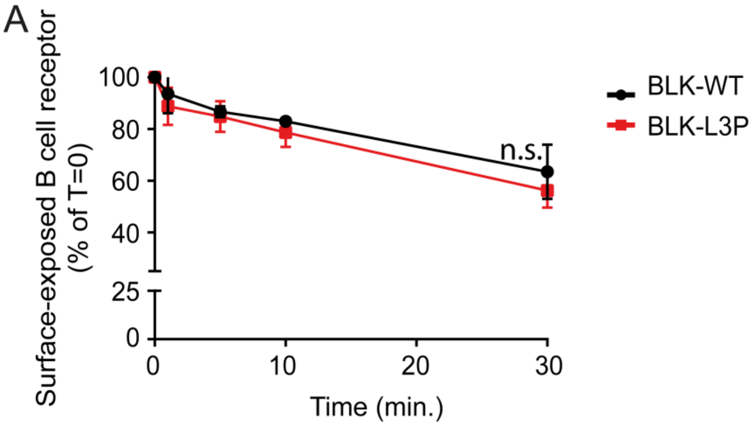

Figure S2: Ligand-induced B cell receptor internalization. (**A**) Surface exposed B cell receptor upon cross-linking with anti-IgG antibodies over time on B-LCLs expressing either L3P- or common BLK variant. *P-value <0.05, **P-value <0.01, Two-tailed Wilcoxon-signed rank test.
